# Depression—an underrecognized target for prevention of dementia in Alzheimer’s disease

**DOI:** 10.1038/s41398-020-0839-1

**Published:** 2020-05-20

**Authors:** Forugh S. Dafsari, Frank Jessen

**Affiliations:** 1 Department of Psychiatry and Psychotherapy, University of Cologne, Faculty of Medicine and University Hospital Cologne, Kerpener Straße 62, 50937 Cologne, Germany; 2 Max-Planck-Institute for Metabolism Research, Gleueler Str. 50, 50931 Cologne, Germany; 3 German Center for Neurodegenerative Disease (DZNE), Sigmund-Freud-Str. 27, 53127 Bonn, Germany

**Keywords:** Predictive markers, Pathogenesis, Diagnostic markers, Depression

## Abstract

It is broadly acknowledged that the onset of dementia in Alzheimer’s disease (AD) may be modifiable by the management of risk factors. While several recent guidelines and multidomain intervention trials on prevention of cognitive decline address lifestyle factors and risk diseases, such as hypertension and diabetes, a special reference to the established risk factor of depression or depressive symptoms is systematically lacking. In this article we review epidemiological studies and biological mechanisms linking depression with AD and cognitive decline. We also emphasize the effects of antidepressive treatment on AD pathology including the molecular effects of antidepressants on neurogenesis, amyloid burden, tau pathology, and inflammation. We advocate moving depression and depressive symptoms into the focus of prevention of cognitive decline and dementia. We constitute that early treatment of depressive symptoms may impact on the disease course of AD and affect the risk of developing dementia and we propose the need for clinical trials.

## Introduction

Dementia is one of the most prevalent brain disorders in old age. It is among the leading causes of disability worldwide and a significant public health concern. Alzheimer’s disease (AD) is the most common cause of dementia. Dementia negatively impacts quality of life, global functioning, physical health and leads to a significant increase in morbidity and mortality^1^. An estimated 47 million people worldwide were living with dementia in 2015 and this number is expected to double every 20 years to almost 132 million in 2050^2,3^. Although some of the dementia symptoms are treatable for a restricted period of time, it can neither be cured nor substantially stabilized over time at present. Nevertheless, the onset of dementia might be modifiable by the management of potential risk factors. The regional distribution of new dementia cases in 2015 represents an increased proportion of new cases in Asia, the Americas and Africa, while the proportion in Europe has decreased. A decline in age-specific incidence of dementia in high income countries might arise from changes in risk-reduction strategies implemented in these countries^3^.

Growing evidence implies that depression, which is a treatable condition, is a risk factor for dementia. In a life-course model of contribution of modifiable risk factors to dementia, the elimination of depression is calculated to produce a 4% reduction in dementia incidence on the population level, exceeding the estimated effects of hypertension (2%), diabetes (1.2), obesity (0.8%), and any physical inactivity (2.6%)^4^. However, recognition of depression in the context of dementia prevention has so far not been attained. The recently published WHO guidelines on risk reduction of cognitive decline and dementia concludes that there is compelling evidence for the association of depression and dementia risk, but data on the effectiveness of depression treatment in reducing dementia risk are missing, which prevents recommending treatment of depression in the prevention of cognitive decline^5^. A better understanding of the role of depression as a risk factor for dementia is crucial in this debate, as it may constitute a promising target for prevention strategies.

## Depression and the risk of developing dementia

A substantial number of epidemiological studies have linked depression to cognitive decline and dementia. The inter-relationship between these clinical entities is complex and not conclusively understood.

### Depression as a risk factor for dementia

The first large population-based studies over two decades ago and subsequent epidemiological studies identified depression as a factor increasing the risk for cognitive decline and development of dementia, particularly dementia in AD^6–9^. Growing evidence from meta-analyses suggested that depression is associated with a more than twofold increase in dementia risk^10,11^, indicating a causal factor hypothesis. Evidence from longitudinal studies confirms a graded association between the severity of depressive symptoms and the risk of dementia, with the risk being more pronounced in severe depression^12^. In addition, studies suggest a strong link between the number of depressive episodes and the risk of developing dementia, indicating a 14% increase in risk for all-cause dementia with each episode of depression^13,14^.

### Depression as an early sign or prodrome of dementia

Additional studies have shown that depressive symptoms before the onset of AD are significantly associated with the development of AD dementia, even when the onset of depressive symptoms occurred more than 25 years before the onset of cognitive symptoms^15^. Because the neurodegenerative changes in AD precede the clinical diagnosis by several years, depressive symptoms may therefore also be one of the earliest non-cognitive manifestations of this neurodegenerative disease, suggesting a reverse causality hypothesis^16,17^.

Moreover, the timing of depression may be important in defining the nature of the association between depression and dementia^18^. A 28-year follow-up cohort study found that depressive symptoms later in life were significantly associated with the development of dementia, while depressive symptoms earlier in the study were not. The results suggest that depressive symptoms might be a prodromal feature of dementia^19^. Late life depression in particular has been associated with an increased risk for all-cause dementia, vascular dementia (VaD) and AD^20^. Evidence from retrospective cohort studies suggests that the risk of AD is doubled in individuals with depressive symptoms in late life (alone or in combination with midlife symptoms), whereas the risk of VaD is more than tripled in those with midlife and late life depression^21^. Studies with repeated measures of depressive symptoms indicate that the subsequent risk of dementia differs with different courses of depression. The higher risk of dementia could only be found in the increasing trajectory of depressive symptoms, further suggesting depression might be a prodrome of dementia^22–24^.

### Depression as an accelerating factor of cognitive decline before and within dementia

Dementia and mild cognitive impairment (MCI) have been related to an increased risk of depressive symptoms. The prevalence of depression in MCI patients is high and depression is the predominant neuropsychiatric symptom of amnestic MCI^25^. In a recent meta-analysis, the overall pooled prevalence of depression in MCI patients was 32%^26^. The overall prevalence of depressive disorders among dementia patients is estimated to be 25–30% with a significantly higher prevalence of depressive disorders in VaD (40–50%) and unspecified dementia (32%) compared with AD (up to 20%)^27–30^. Moreover, numerous studies have shown the potential role of depression in the conversion from normal cognition to MCI and from MCI to dementia. The majority of studies found, depression is an important accelerating factor contributing to the progression and conversion from a cognitively normal state to MCI and dementia^8,17,31–33^. Older adults with a combination of MCI and recently active depression are a particularly high-risk subgroup^34^.

**Fig. 1:**
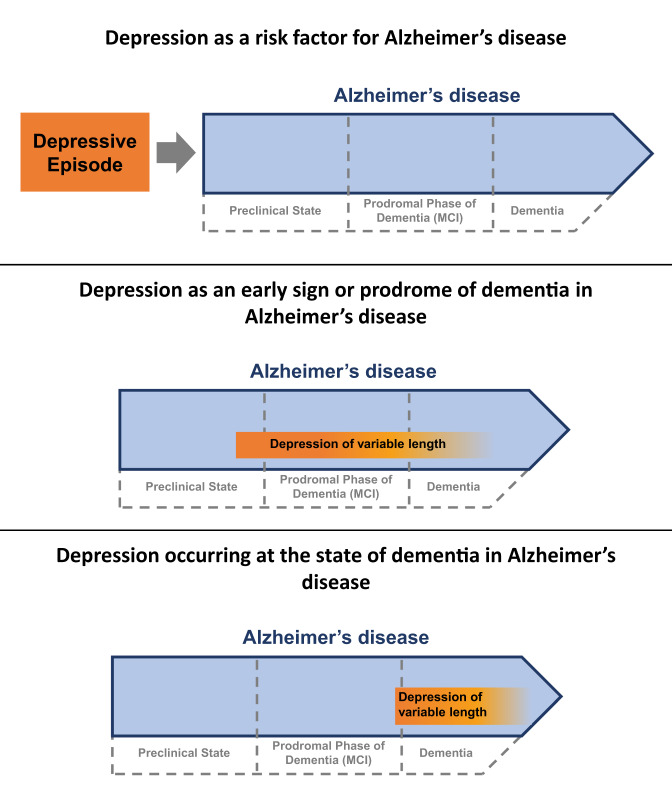
The relationship between depression and cognitive decline throughout the development and clinical course of Alzheimer‘s disease (AD). Depression can occur in three different stages in relation to the process of neurodegeneration in AD. Depression can be a predisposing risk factor occuring before the onset of AD pathology. It might also be an early sign of neurodegenerative changes or a prodromal symptom with or without cognitive deficits. Finally, it may occur at the more advanced dementia stage of AD. In every stage depression is an important accelerating factor contributing to the clinical progression and conversion from a preclinical state to MCI and to dementia.

Thus, the association between depression and dementia has led to an ongoing debate on the underlying reasons and the direction of causation (Fig. 1). There is considerable research indicating that depression is a true predisposing risk factor for dementia. Depression might also be an early prodromal symptom, an early sign of neurodegenerative changes that occur in dementia, a psychological reaction to cognitive and functional disability (“cognitive burden”), or a symptom of a related risk factor (confounder), such as cerebrovascular disease^35,36^. However, each of these associations suggests that depression is a potential modifiable factor for the development of dementia and treatment of depression might have the potential to delay dementia progression.

## Biological mechanisms linking depression to dementia

The most prominent biological mechanisms linking depression to dementia include a hypothalamic-pituitary-adrenal axis dysregulation with alterations in glucocorticoid steroid levels, hippocampal atrophy, inflammatory changes, deficits of nerve growth factors, increased deposition of Amyloid-β (Aβ) plaques and cerebrovascular disease.

### Hypothalamic-pituitary-adrenal axis dysregulation

**Fig. 2:**
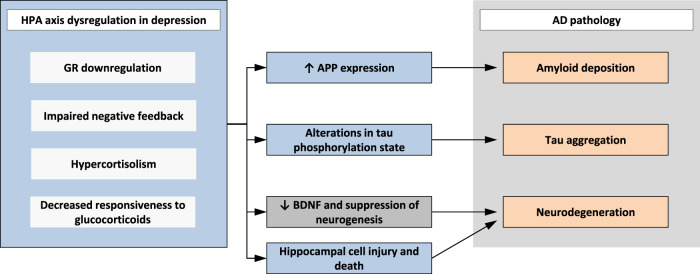
Impact of HPA axis dysregulation in depression on AD pathology. HPA axis: hypothalamic-pituitary-adrenal axis, GR: glucocorticoid receptor, APP: amyloid precursor protein, BDNF: brain-derived neurotrophic factor, AD: Alzheimer’s disease.

The association between depression and dementia may emerge from the impact of depression on the hypothalamic-pituitary-adrenal axis (HPA axis), resulting in chronic elevation of adrenal glucocorticoids and impaired negative feedback of the HPA axis (Fig. 2). It is well known that depression is associated with a dysregulation of the HPA axis, a downregulation of glucocorticoid receptors in the hypothalamus and pituitary gland. This results in a decreased responsiveness to glucocorticoids and impaired negative feedback regulation^37^. The “glucocorticoid cascade hypothesis” states that glucocorticoids participate in a feed-forward cascade of effects on the brain and body, leading to progressive glucocorticoid-induced neurotoxicity and promoting progressive elevation of adrenal steroids and dysregulation of the HPA axis^38–40^. Glucocorticoids have been reported to increase amyloid precursor protein (APP) expression, tau accumulation and lead to alterations in tau phosphorylation state in animal studies^41–43^. Elevation of glucocorticoids can cause damage to brain structure and function particularly in the hippocampus^44,45^. Structural changes in the hippocampus underlie the pathophysiology of dementia and major depressive disorder (MDD). Evidence suggests that HPA axis hyperactivity may not only be an early event in the course of AD but also a factor contributing to further cognitive decline, accelerating disease progression, and clinical worsening over time^46–48^.

### Hippocampal atrophy

*Hippocampal atrophy* is one of the main and early brain changes in AD. Evidence from structural imaging studies suggests that depression in late life is associated with a reduced hippocampal volume relative to non-depressed elderly control subjects^49,50^. The hippocampus plays a crucial role in the brain’s response to psychosocial stress by serving both as a target and a regulator of the brain’s response to stress^51^. Hippocampal neurons express glucocorticoid receptors and hippocampal inhibitory afferents suppress and regulate the release of hypothalamic corticotropin-releasing factor (CRF)^51^. Prolonged hypercortisolemia may promote hippocampal cell injury and death, hippocampal atrophy and cognitive decline^52^. Stress-induced structural remodeling in the adult hippocampus includes suppression of neurogenesis as well as debranching and shortening of dendrites as main cellular mechanisms in the impairment of neural plasticity in the human hippocampus. Increased loss of hippocampal volume has consistently been found to correlate with the duration of depression^18^. Thus, it has been hypothesized that long-term exposure to stress or depression leads to a smaller hippocampus, contributing to the development of dementia^18,50,53^.

### Inflammation

**Fig. 3:**
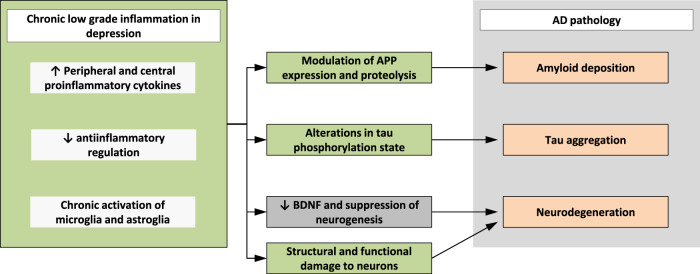
Impact of inflammation in depression on AD pathology. APP: amyloid precursor protein, BDNF: Brain-derived neurotrophic factor, AD: Alzheimer’s disease.

Depression and dementia share a closely linked *inflammatory etiology* (Fig. 3). A significant proportion of depressed patients exhibit chronic, low-grade inflammation and numerous studies have reported increases in circulating peripheral and central pro-inflammatory cytokines (IL-1β, IL-6, TNF-α), inflammatory mediators, acute-phase reactants (CRP) and a decrease in anti-inflammatory regulation in depressed patients^54–57^. Low-grade chronic inflammation has been shown to decrease 5-hydroxytryptamine (5-HT) and dopamin (DA) synthesis in brainstem nuclei as well as reduce synaptic availability and release of monoamines in the brain^58–60^. This potentially leads to an undersupply of cortical regions with monoaminergic neurotransmitters, which is a fundamental mechanism in the pathophysiology of depression.

Furthermore, pathologies implicated in the progression of depression include loss of astroglia, loss of somatostatin-positive interneurons, and chronic *microglial activation*^61,62^. In a ^18^F-FEPPA PET study in MDD patients investigating microglial activation, as measured by translocator protein (TSPO) total distribution volume (VT) throughout grey-matter regions, greater levels of microglial activation were associated with greater duration of untreated MDD. The total duration of illness predicted greater microglial activation and duration of antidepressant exposure was a similar magnitude negative predictor of microglial activation. These findings implicate that microglial activation becomes progressive in MDD when untreated^61^. As chronic microglial activation is considered to be involved in the progression of neurodegenerative disease, it might play a crucial role in the inter-relationship between depression and dementia. Emerging evidence suggests that microglial activation has a causal role in the pathogenesis of the two most common dementia diseases, AD and VaD^63,64^. AD patients have activated microglia in the brain and high circulating inflammatory markers including TNF-α, IL-1β, IL-6, TGF-β, IL-12 and IL-18 and higher CSF concentrations of TGF-β^64–66^. The role of neuroinflammation is strengthened by the finding that genes for immune receptors and protein-coding changes in genes highly expressed in microglia such as TREM2, CD33, PLCG2 and ABI3 are associated with AD^63,67^. These genetic findings provide additional evidence that the microglia-mediated innate immune response contributes directly to the development of AD^67^. Pathological accumulation of Aβ is a key factor that drives neuroinflammation in AD, as the exposure of microglia to preaggregated Aβ_1–42_ increases production of pro-inflammatory cytokines^63,64^. Current evidence from clinical studies suggests that several inflammatory markers in serum are associated with the risk of all-cause dementia in community-dwelling older adults^64^. The risk for conversion from MCI to the dementia stage of AD is increased in patients with elevated concentrations of pro-inflammatory cytokines and decreased anti-inflammatory cytokines^63^.

### Nerve growth factors

Cytokines activate inflammatory signaling pathways in the brain resulting in a decrease in growth factors such as brain-derived neurotrophic factor (BDNF). Thereby, they do not only have the capability of influencing neurocircuitry systems to produce behavioral alterations but also to reduce synaptic plasticity and neurogenesis^56^. BDNF has an essential role in neurogenesis, synaptogenesis and synaptic plasticity and is involved in neuroprotective and neuroregenerative processes. Both depressed as well as AD patients have impaired BDNF signaling pathways. There is a high degree of interaction between BDNF and the serotonergic system^68^. The expression of BDNF is stimulated by 5-HT via cAMP-response-element-binding protein (CREB), and BDNF enhances the growth and survival of 5-HT neurons^69^. Moreover, polymorphisms in the BDNF gene are associated with a significantly reduced hippocampal volume in depressed patients^68^. Both depressed and AD patients demonstrate decreased messenger RNA levels of BDNF in the hippocampus^18^.

### Noradrenalin, serotonine, amyloid plaques, and neurofibrillary tangles

**Fig. 4:**
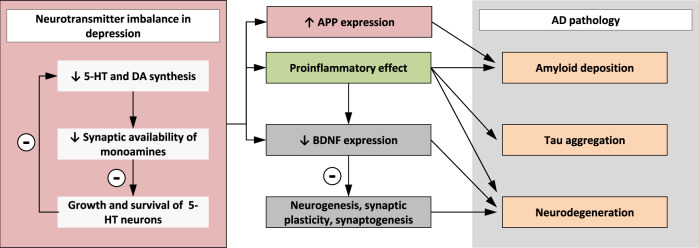
Impact of neurotransmitter imbalance in depression on AD pathology. 5-HT: 5-Hydroxytryptamine, DA: Dopamin, BDNF: Brain-derived neurotrophic factor, AD: Alzheimer’s disease.

Amyloid plaques and neurofibrillary tangles are neuropathologic hallmarks of AD. Furthermore, modifications in the serotonergic and noradrenergic system are involved in the pathogenesis of AD and depression (Fig. 4). Noradrenalin is a neurotransmitter of major importance in the pathophysiology and treatment of depressive disorders. In addition to its role as a neurotransmitter, it has potent anti-inflammatory, neurotrophic and neuroprotective effects as well as effects on amyloid deposition. Lesions of the Locus coeruleus (LC) in mouse models of AD led to increased inflammation, neuronal damage and increase in Aβ plaque burden. LC degeneration compromised microglial migration and Aβ phagocytosis in vivo, suggesting that a loss of noradrenaline increases inflammation and Aβ deposition^63^. The serotonergic system also influences the production of Aβ and therefore provides another link between depression and AD. Serotonin increases the release of non-amyloidogenic APP via 5-HT_2A_ and 5-HT_2C_ receptors, thereby disfavoring the formation of neurotoxic Aβ. A reduction in 5-HT levels could potentially alter the cleavage of APP facilitating Aβ production^68^. Another hypothesis linking depression to Aβ implies an increase of Aβ production by a stress response associated with depression and including glucocorticoid levels^18^. Animal models of AD indicate that stress-level glucocorticoid administration increases Aβ formation by increasing steady-state levels of APP and β-secretase enzyme (BACE). Higher brain Aβ burden was associated with increasing anxious-depressive symptoms in cognitively normal older adults and supports the hypothesis that emerging neuropsychiatric symptoms might represent an early manifestation of preclinical AD^70^. Recent evidence suggests that cortical amyloid moderates the association between worsening depressive symptoms and declining cognition in older adults. Changes in depression and cognition among older adults with higher cortical amyloid might suggest that depressive symptoms may serve as targets in delaying the clinical symptoms of Alzheimer disease^71^.

Additionally, glucocorticoids augment tau accumulation, indicating that it may accelerate the development of neurofibrillary tangles^41^. Results from tau-PET imaging in cognitively normal older adults suggest an association between depressive symptoms and tau in the entorhinal cortex and inferior temporal cortex^72^. A newly emerging hypothesis constitutes, that sleep disturbances and the disruption of circadian rhythms is associated with amyloid deposition, tau hyperphosphorylation and aggregation, neuronal and synaptic dysfunction and degeneration^73,74^. A bidirectional association between sleep disturbances and neurodegeneration has been proposed with disturbed sleep contributing to Aβ accumulation and vice versa^75^. Self-report of poor sleep is associated with greater brain amyloid burden, as measured by PET with Pittsburgh compound B (PiB) in humans^76^. As sleep abnormalities are common symptoms of depression, they might be a principal component of the causal pathway linking depression to AD pathogenesis and accelerating AD progression.

### Cerebrovascular changes

Additionally, evidence suggests that *cerebrovascular changes* might constitute a link between depression and dementia. The ‘vascular depression hypothesis’ states that cerebrovascular disease may predispose, precipitate, or perpetuate some geriatric depressive syndromes^77^. In support of the hypothesized association between vascular depression and dementia studies have confirmed that ischemic lesions, particularly in frontostriatal brain regions, may lead to cognitive deficits, executive dysfunction and psychomotor retardation. Vascular disease and the metabolic syndrome are associated with a dysregulation of the HPA axis, elevated cortisol levels and increase of pro-inflammatory cytokines^18^. Chronic inflammation increases the risk of vascular events thereby contributing to VaD.

**Fig. 5:**
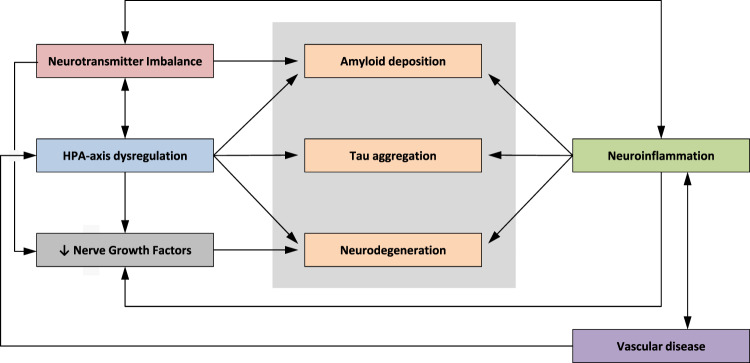
Impact of depression-related mechanisms on AD pathology. AD: Alzheimer’s disease, HPA: Hypothalamic-pituitary-adrenal axis.

In summary, the mechanisms that contribute to the association between depression and dementia are multifactorial. Figure 5 summarizes the impact of depression-related mechanisms on AD pathology.

## Antidepressive treatment and neurogenesis

### Animal studies on antidepressants and neurogenesis

Although depression results in cell atrophy and loss, these effects are reversible upon elimination of stress or with antidepressant treatment. Antidepressants have the potential to increase neurogenesis and to reverse some of the effects of stress, including reductions in dendrite number and length, neurogenesis, gliogenesis, and GABAergic cell loss (Table 1). Studies suggest that antidepressant treatment may have neuroprotective properties by particularly increasing the proliferation of neural progenitors in the subgranulate zone of the hippocampus and gliogenesis (i.e. oligodendrocytes) in the prefrontal cortex^78–81^. Animal studies have shown that stress-induced changes in neural plasticity and structural volume can be prevented by antidepressant treatment. Upregulation of neurogenesis in the adult hippocampus has been demonstrated in rodents after administration of different classes of antidepressants and appears to be dependent on chronic antidepressant treatment^79,82^. Chronic treatment with antidepressants such as the selective serotonin reuptake inhibitor (SSRI) Fluoxetine and the monoamine oxidase inhibitor Moclobemide reversed the stress-induced changes in hippocampal neurogenesis, inhibited apoptosis in hippocampal primary neurons and increased BDNF expression in mice^52,83–90^. Fluoxetin increased the sizes of the hippocampal CA1 and dentate gyrus in a mouse model of AD. Additionally, the synaptic plasticity of neurons in the hippocampus was remodeled, and the expression levels of synaptic-related proteins were increased along with activation of the CREB protein/BDNF signaling pathway^91^. A reversal of structural remodeling may be a desirable goal for antidepressant therapy in dementia prevention.

**Table 1:** Summary of antidepressant actions with neuroprotective effects.

Mechanism	Neuroprotective effect of individual compounds or drug classes
**Inflammation**	▪ Antidepressants reduce peripheral pro-inflammatory markers and increase anti-inflammatory cytokines^a^
	▪ SSRIs limit microglial and astroglial inflammatory activation (e.g. TNF-α-, NO production)
	▪ Fluoxetine promotes downregulation of genes involved in pro-inflammatory pathways (e.g. IL-6, NF-κb, TNF and acute-phase response signaling)^b^
	▪ Bupropion lowers production of TNF-α and IFN-γ^b^
	▪ Venlafaxine augments TGF-β release, reduces secretion of IL-6, IFN-γ and changes microglial phenotype from activated to resting morphology^b^
	▪ Moclobemide exerts anti-inflammatory effects by affecting the balance between pro- and anti-inflammatory cytokines (IL-1β, TNF-α/IL-10)^b^
**Neurotransmitter metabolism**	▪ Antidepressants influence monoamine metabolism and increase levels of 5-HT and NA^a^
	▪ NA has anti-inflammatory, neurotrophic and neuroprotective effects
	▪ NA influences microglial migration, Aβ phagocytosis and effects amyloid deposition
	▪ 5-HT increases release of non-amyloidogenic APP via 5-HT2A and 5-HT2C receptors thereby disfavoring the formation of neurotoxic Aβ
**HPA axis and neurogenesis**	▪ Antidepressants increase neurogenesis, reverse reduction in dendrite number/length and GABAergic cell loss^b^
	▪ increase the proliferation of neural progenitors in the subgranulate zone of the hippocampus and gliogenesis (i.e. oligodendrocytes) in the prefrontal cortex
	▪ increase BDNF transcription by BDNF-TrkB signaling, MAPK and PI3K pathways
	▪ effect Wnt-GSK-3 and influence growth and guidance of neurons and dendritic arborization
	▪ Fluoxetine and Moclobemide reverse stress-induced changes in hippocampal neurogenesis, inhibit apoptosis in hippocampal primary neurons and increase BDNF expression^b^
	▪ Fluoxetin increases the sizes of hippocampal CA1 and dentate gyrus, remodels synaptic plasticity of neurons in the hippocampus (activation of CREB protein/BDNF signaling pathway)^b^
**Amyloid-β**	▪ Antidepressants reduce amyloid plaque burden by shifting the balance from pro- toward non-amyloidogenic APP processing^a^
	▪ Antidepressants up-regulate cAMP cascade in hippocampus and cerebral cortex leading to enhancement of synaptic plasticity^b^
	▪ SSRIs reduce ISF Aβ levels in animal models and CSF Aβ concentrations in humans
	▪ Citalopram suppresses generation of Aβ and decreases levels of insoluble Aβ 40 in hippocampal and cortical tissue
	▪ Fluoxetine prevents the increase of Aβ accumulation
	▪ Tranylcypromine prevents Aβ-induced neuronal death and Aβ aggregation^b^
	▪ Amitriptyline inhibits Aβ1–42-induced activation of ERK1/2 and exerts neuroprotective effects against Aβ1–42-induced neurotoxicity^b^
**Tau**	▪ Escitalopram ameliorates forskolin- and Aβ1–42-induced tau hyperphosphorylation in primary hippocampal neurons through activation of PKA and 5-HT1A receptor mediated Akt/GSK-3β pathway^b^

*SSRIs* selective serotonin reuptake inhibitors, *TNF-α* tumor necrosis factor alpha, *NO* nitric oxide, *IL-6* Interleukin 6, *NF-κb* nuclear factor kappa-light-chain-enhancer of activated B cells, *IFN-γ* interferon gamma, *TGF-β* transforming growth factor-β, *IL-1β* Interleukin 1 beta, *IL-10* Interleukin 10, *5-HT* 5-hydroxytryptamine, *NA* noradrenalin, *APP* amyloid precursor protein, *HPA axis* hypothalamic-pituitary-adrenal axis, *BDNF* brain-derived neurotrophic factor, *CREB* cAMP-response element-binding protein, *BDNF-TrkB* brain-derived neurotrophic factor tropomyosin related kinase B, *MAPK* mitogen-activated protein kinase, *PI3K* phosphatidyl inositol 3-kinase-Akt pathways, *Wnt-GSK-3* Wnt-glycogen synthase kinase-3, *ERK1/2* extracellular signal-regulated kinase 1 and 2, *PKA* protein kinase A.

^a^In animal and human studies.

^b^In animal studies.

The focus of the neurotrophic hypothesis has been on BDNF, although there is also evidence that antidepressants increase VEGF (vascular endothelial growth factor), FGF2 (fibroblast growth factor 2) and IGF-1 (insulin growth factor-1)^81,82^. Chronic stress results in decreased transcription of BDNF, whereas chronic treatment with monoamine modulators results in increased BDNF transcription^51^. BDNF-TrkB (tropomysin related kinase B) signaling, involved in birth and survival of neurons in the hippocampus, stimulates several signaling cascades including mitogen-activated protein kinase (MAPK) and phosphatidyl inositol 3-kinase-Akt pathways (PI3K). A decreased MAPK function is involved in the pathophysiology of depression and antidepressants produce an enhanced MAPK signaling^92^. Studies indicate that BDNF is a key factor for antidepressant effects, which are partly mediated by induction of neuronal and glial cell birth and possibly also by effects on dendrite complexity^81^. There is evidence for Wnt-GSK-3 (glycogen synthase kinase-3) in the actions of antidepressants. It is important for the growth and guidance of neurons during development and dendritic arborization in adult brain^81^ and might contribute to the reversal of neural atrophy by antidepressants.

### Antidepressants and neurogenesis in humans

Antidepressants may have neuroprotective effects in humans as well and protect against hippocampal volume loss associated with cumulative episodes of depression^93^. Evidence indicates that normalizing 5-HT activity in depression may have specific beneficial effects on cognition^94^. Studies have shown that SSRIs may reduce the risk of AD in depressed individuals. A retrospective study of over 1.4 million individuals over a decade suggested that depressed individuals who were treated with an antidepressant only once or for a short period of time, had a greater risk of AD than those with chronic antidepressant treatment. Continued long-term antidepressant treatment was associated with a reduction in the rate of dementia, however, not to the same level as the rate for the general population^95,96^.

It has also been suggested that treatment with an SSRI may improve cognitive function and daily living in MCI and AD patients^97^. Randomized, placebo-controlled trials evaluating the effect of SSRIs on cognition in MCI patients and in AD reported a favorable and enhancing effect of Fluoxetine on memory and cognition in MCI patients^98^ and Sertraline in AD patients^99,100^.

Few studies investigated the influence of SSRI treatment on the progression from normal cognition to MCI and AD. A retrospective analysis in a population of cognitively healthy adults discharged from psychiatric healthcare service with a diagnosis of depression reported a lower rate of AD in patients receiving first-generation antidepressants compared with SSRI, serotonin-norepinephrine reuptake inhibitors (SNRI) and no treatment^97^. A prospective longitudinal cohort study in non-depressed participants revealed, that in MCI patients with a history of depression, long-term SSRI treatment (>4 years) was significantly associated with a delayed progression to AD by approximately 3 years. Thus, long-term SSRI treatment may delay progression from MCI to AD^36^.

Therefore, long-term continued treatment with antidepressants might promote neurogenesis in the human hippocampus, thereby decrease the risk of developing dementia in cognitively healthy individuals and decelerate progression to dementia in MCI patients.

## Antidepressive treatment and amyloid burden

### Animal studies on antidepressants and amyloid

Animal models have shown that SSRIs may reduce amyloid plaque burden and cognitive impairment, presumably by shifting the balance from pro- toward non-amyloidogenic processing of APP^36^ (Table 1). In mice, brain interstitial fluid (ISF) Aβ levels were decreased significantly following administration of SSRI and direct infusion of serotonin into the hippocampus reduced ISF Aβ levels^96,101^. Serotonin signaling suppresses not only the generation of Aβ in vitro but also in animal models of AD^101^. The chronic treatment with Citalopram arrested the growth of preexisting plaques and caused a 50% reduction in brain plaque load in mice by significantly decreasing the levels of insoluble Aβ 40 in hippocampal and cortical tissues^96,101,102^. In addition, Fluoxetine applied to a mouse model of AD (3xTg AD mice) has the potential to effectively prevent the increase of Aβ accumulation^86,91^. Studies indicate that the MAO inhibitor tranylcypromine protects cortical neurons challenged with synthetic Aβ1–42 oligomers. Tranylcypromine significantly prevents Aβ-induced neuronal death and influences the early events of the Aβ aggregation process in a concentration-dependent manner^103^. Finally, studies of the pharmacology of antidepressants suggest that pre-treatment with amitriptyline may have the capability to modify the epigenetic status and induce gene expression changes associated with neuronal cell death. Amitriptyline inhibits Aβ1–42-induced activation of extracellular signal-regulated kinase 1 and 2 (ERK1/2) and exerts neuroprotective effects against neurotoxicity induced by Aβ1–42. ERK1/2 control intracellular processes such as cell growth, differentiation, survival and cell death^104^. Amyloid peptide deposition interferes with CREB phosphorylation^82^. Recent studies demonstrate that chronic antidepressant treatment up-regulates the cAMP cascade in hippocampus and cerebral cortex, including increased particulate levels of cAMP-dependent protein kinase (PKA), upregulation of the function and expression of CREB, thereby leading to an enhancement of synaptic plasticity^82,91^.

### Antidepressants and amyloid in human studies

Data from human studies suggest that serotonin signaling is associated with less Aβ accumulation. In a retrospective study antidepressant-treated cognitively normal elderly participants had significantly less amyloid load as quantified by PET imaging with PIB compared to participants who were not exposed to antidepressants within the past 5 years. Cumulative time of antidepressant use within the 5-year period preceding the scan correlated with less plaque load^96^. In a prospective study in healthy human volunteers, the effects of citalopram on Aβ production and Aβ concentrations in CSF were measured with CSF sampling during acute dosing of citalopram. Aβ production in CSF was slowed in the citalopram group compared to placebo and the change was associated with an almost 40% decrease in total CSF Aβ concentrations in the antidepressant-treated group^101^.

These results suggest that inhibition of Aβ oligomer-mediated aggregation significantly contributes to the overall neuroprotective activity of antidepressants. The ability to decrease Aβ concentrations by antidepressant treatment is an important potential strategy for AD and might be a key target for future AD prevention.

## Antidepressive treatment and tau pathology

Tau hyperphosphorylation is one of the main pathological features of AD. Evidence from animal studies suggests that the SSRI escitalopram ameliorates forskolin-induced tau hyperphosphorylation and spatial memory impairment in rats. It was shown that escitalopram may protect tau from hyperphosphorylation induced by pharmacological activation of protein kinase A (PKA). These effects of escitalopram do not occur via an anti-anxiety activity but involve the Akt/GSK-3β signaling pathway^105^. To investigate whether escitalopram could inhibit Aβ-induced tau hyperphosphorylation and the underlying mechanisms, rat primary hippocampal neurons were treated with Aβ1-42 and the effect of escitalopram on tau hyperphosphorylation was examined^106^. Escitalopram decreased Aβ1-42-induced tau hyperphosphorylation and activated the Akt/GSK-3β pathway. It improved Aβ1-42 induced impairment of neurite outgrowth and spine density, and reversed Aβ1-42 induced reduction of synaptic proteins. These results indicate that escitalopram might have the potential to attenuate Aβ1-42-induced tau hyperphosphorylation in primary hippocampal neurons through the 5-HT_1A_ receptor mediated Akt/GSK-3β pathway^106^.

However, the few currently available data from animal studies and the lack of human studies are insufficient to specify the underlying mechanisms and effects of antidepressants on tau pathology. Antidepressant treatment may be a promising strategy in the attenuation of tau pathology. Further research is needed in order to investigate the relationship between antidepressant treatment and tau pathology in humans.

## Effects of antidepressants on inflammation

### Animal studies on antidepressants and inflammation

The induction of significant molecular changes by chronic stress leading to the increase in pro-inflammatory cytokines is generally reversed by antidepressants such as fluoxetine, imipramine, and tianeptine. Findings suggest that antidepressants possess significant anti-inflammatory properties^107–109^ (Table 1). In addition to their effects on cells of the peripheral immune system, SSRIs can limit microglial and astroglial inflammatory processes^108^. Fluoxetine acts on neurons and promotes the downregulation of genes involved in pro-inflammatory response pathways (e.g. expression of IL-6 signaling, NF-κb signaling, acute-phase response signaling) and of TNF signaling-related molecules in rats^110^. Further, the dopamine enhancer bupropion is also known to inhibit pro-inflammatory cytokine production and to lower production of TNF-α and Interferon-γ in mice^111^. Studies indicate that SSRIs (e.g. fluoxetine, paroxetine, sertraline) potently inhibit microglial TNF-α and NO production. SNRIs such as venlafaxine as well as the MAO inhibitor moclobemide possess anti-inflammatory properties in mixed glial cultures^112,113^. Selective noradrenaline reuptake inhibitors, which increase endogenous noradrenaline concentrations, can reduce neuroinflammation and partly rescue microglial functions^63^.

### Antidepressants and inflammation in human studies

In a 18F-FEPPA PET study microglial activation was greater in MDD patients with long periods of no antidepressant treatment than in MDD patients with short periods of no antidepressant treatment. Consistently, the yearly increase in microglial activation was no longer evident when antidepressant treatment was given^61^. The specific biological mechanisms of SSRI to modulate the microglial inflammatory response have not yet been fully understood. A variety of possible mechanisms have been researched such as mediated effects via 5-HTT, cyclic adenosine monophosphate signaling, transcription factor NF-κB, and the anti-inflammatory cytokine IL-10^108,109^.

Since depression and dementia are both associated with an inflammatory etiology and the risk for conversion from MCI to the dementia stage of AD is increased in patients with elevated concentrations of pro-inflammatory cytokines, early antidepressant treatment might be an essential strategy in dementia prevention.

## Conclusions and future perspective

Dementia in AD has long been considered to be not preventable. Recent evidence implicates a considerable progress and reveals that a substantial fraction of dementia cases might indeed be preventable. Treatment of depression as a modifiable risk factor may contribute to prevention and delay of dementia. Although most dementia cases are diagnosed later in life, dementia has a long preclinical phase. Particularly AD pathology develops earlier and has already been ongoing for decades when the patients express the first cognitive symptoms^114,115^. In cognitively normal subjects, amyloid positivity as determined by PET or CSF findings precedes the onset of the symptoms by 20–30 years^116^. Selecting and treating patients with cognitive deficits or functional disability may be too late because amyloid burden is already extensive. The main objective of preclinical AD detection is to identify individuals at high risk of progression to AD, in order to enable early treatment and thereby prevent cognitive decline or facilitate a delay of cognitive symptoms. Prevention or delay of dementia onset is a public health priority with the potential to reduce the disability of individuals and the associated societal and economic burden^4^.

It is not only epidemiologically but also biologically plausible that depression increases dementia risk. Antidepressant treatment of depressive symptoms affects the risk, development and progress of dementia. Evidence suggests that antidepressant treatment promotes neurogenesis in the human hippocampus and inhibits Aβ oligomer-mediated aggregation. It might also be a promising strategy in the attenuation of tau pathology. Furthermore, antidepressant treatment possesses significant anti-inflammatory properties and limits microglial and astroglial inflammatory processes which play a causal role in the pathogenesis of dementia. Therefore, it is consequently a conceivable hypothesis that long-term continued treatment with antidepressants may decrease the risk of developing dementia not only among individuals with severe and recurrent depression but also in less severe depressive disorders (mild to moderate depression) and subsyndromal depression in individuals at high risk for AD.

Early identification of depressive symptoms, including subclinical features and subsequent antidepressive treatment may be essential in the prevention of dementia in order to reduce neurotoxic effects of depressive episodes. It is crucial to improve the awareness in the research and healthcare community that depressive symptoms are a risk factor and may be an early sign of future cognitive decline. A proposal for next steps in further research on depressive symptoms in the context of AD prevention is summarized in Table 2. The detection of subsyndromal depression in individuals with an increased risk of AD, including carriers of the apolipoprotein E (*APOE*) ε4 allele, family history of AD or amyloid pathology with no or only subtle cognitive impairment, is crucial and could generate an opportunity for early intervention of dementia. Future studies should carry out longitudinal assessments to determine the effect of antidepressive treatment on conversion to dementia in cognitively healthy individuals with depressive disorders or subsyndromal depression and in MCI patients. The course of depressive symptoms over a lifetime varies across individuals and should be taken into account. The remitting and relapsing nature of depression necessitates studies on the course of depression in relation to risk of dementia and the effects of antidepressant treatment on dementia progression in different depression trajectories. Different trajectories of depressive symptoms might not only predict dementia risk differentially but also determine the effect of antidepressant treatment on the development of dementia. Further, the analysis of baseline cognitive status, medical comorbidities, and the differentiation between late-onset depression (first episode after age 60) and early onset depression (first episode in early or midlife) that recur or continue into later life is essential. A number of studies have been carried out as retrospective analysis with methodological inconsistencies (varying definitions of MCI, dementia and depression) and small sample size. Prospective longitudinal and controlled studies investigating the association between antidepressants and dementia need to follow a large number of patients with different depression severities and trajectories, with and without antidepressant treatment for at least 5–10 years. The treatment of depression as a modifiable risk factor of dementia is inevitable and more research in this field is necessary, as it might contribute to prevention and delay of dementia.

**Table 2:** Next steps for further research on depressive symptoms in the context of AD prevention.

▪ Delineation of symptoms of depression including subclinical symptoms, which specifically reflect initial signs of AD as opposed to symptoms of depression, which are unrelated to AD
▪ Prospective studies with antidepressive treatments and cognitive decline and dementia as primary outcomes
▪ Characterizing mechanisms of action of antidepressive compounds in the early stage of AD, including human studies (e.g. PET)
▪ Improvement of awareness in the larger research and eventually healthcare community that depressive symptoms are a risk factor and may be an early sign of future cognitive decline

*AD* Alzheimer’s disease, *PET* Positron-emission tomography.
